# Electromyographic Evaluation of the Shoulder Muscle after a Fatiguing Isokinetic Protocol in Recreational Overhead Athletes

**DOI:** 10.3390/ijerph18052516

**Published:** 2021-03-03

**Authors:** Sebastian Klich, Adam Kawczyński, Bogdan Pietraszewski, Matteo Zago, Aiguo Chen, Małgorzata Smoter, Hamidollah Hassanlouei, Nicola Lovecchio

**Affiliations:** 1 Department of Paralympic Sport, University School of Physical Education in Wrocław, 51-617 Wrocław, Poland; adam.kawczynski@awf.wroc.pl; 2 Department of Biomechanics, University School of Physical Education in Wrocław, 51-617 Wrocław, Poland; bogdan.pietraszewski@awf.wroc.pl; 3 Dipartimento di Elettronica, Informazione e Bioingegneria, Politecnico di Milano, 20133 Milano, Italy; matteo2.zago@polimi.it; 4 College of Physical Education, Yangzhou University, Yangzhou 225009, China; agchen@yzu.edu.cn; 5 Chinese—Polish Laboratory on Sport and Brain Science, Yangzhou University, Yangzhou 225009, China; 6 Gdańsk Academy of Physical Education and Sport, 80-001 Gdańsk, Poland; m.h.smoter@gmail.com; 7 Department of Cognitive and Behavioral Sciences and Technology in Sport, Shahid Beheshti University, Tehran 1983969411, Iran; hamidhasanlooie@gmail.com; 8 Department of Public Health, Experimental and Forensic Medicine, University of Pavia, 27100 Pavia, Italy; nicola.lovecchio@unipv.it

**Keywords:** overhead sport, SEMG, shoulder girdle, isokinetic, fatigue

## Abstract

The goal of our study was to examine the muscle activity of the shoulder girdle after isokinetic fatigue, which may simulate muscle activities commonly occurring during specific sport-related activities in recreational overhead asymptomatic athletes. We hypothesized that exercise-induced fatigue, reported after isokinetic protocols, may cause a decrease in the median frequency (MF) of the upper trapezius (UT), infraspinatus (IS), and deltoid muscles. Twenty-four male overhead volleyball (*n* = 8), handball (*n* = 8), and tennis (*n* = 8) athletes participated in this study. All subjects were without shoulder injury history. The surface electromyography (SEMG) was collected on the right (dominant) side of the shoulder girdle muscles in the following order: UT, IS and anterior (DA), and posterior deltoideus (DP). The fatigue protocol consisted of three sets of 32 maximum isokinetic concentric contractions while performing shoulder internal and external rotation at an isokinetic speed of 120 o/s. The resultant difference in median frequency (ΔMF) values consistently dropped after the fatiguing tasks across all recorded muscles, in terms of the initial MF (MF_INI_ = 65.1 ± 1.1 Hz) and final MF (MF_FIN_ = 57.9 ± 0.9 Hz), and the main effect of time was significant (F(1,22) = 43.15, *p* < 0.001). MF values decreased mostly for IS (ΔMF_IS_ = −9.9 ± 1.6 Hz) and DP (ΔMF_PD_ = −9.5 ± 1.9 Hz) muscles, while DA and UT showed smaller changes (ΔMF_DA_ = −6.9 ± 1.5 Hz) and (ΔMF_UT_ = −3.2 ± 1.3 Hz). The results of our study show a meaningful contribution in determining increased fatigue of the shoulder girdle muscles during repeated isokinetic internal-external rotation protocols. We have also demonstrated a significant decrease in MF in all examined muscles, especially IS and DA.

## 1. Introduction

Overhead performance requires integration between shoulder mobility and stability connected with neuromuscular control [1]. The characteristics of basic skills in overhead athletes are based on a high rotational velocity generating maximal force about the glenohumeral joint [2]. Any disturbance of either of these factors may lead to an increased risk of shoulder injury [3] caused mainly by chronic muscle fatigue [4] and overloading [5]. Thus, overuse syndrome may result in higher pain sensitivity and functional dysfunction in the shoulder girdle complex [6]. In healthy overhead athletes, increased fatigue may cause scapular dyskinesis [7] by morphological and mechanical muscle-induced alterations in the rotator cuff muscles [3]. Our previous studies have shown multiple changes in the morphological and mechanical properties [3] of the shoulder girdle, as well as alterations in scapulohumeral rhythm and joints’ range of motion [6] following an isokinetic fatigue protocol. Chopp et al. [8] and Noguchi et al. [9] found alterations in scapular rotation and scapular tilt after a fatiguing protocol including arm elevation above the shoulder and internal/external rotation holding. Ebaugh et al. [10,11] found that alterations in scapulothoracic and glenohumeral motion were associated with compensatory motions to minimize the decrease in humeral external rotation. Moreover, it has been found that alterations in scapular orientation, as well as scapulothoracic and glenohumeral motion, may result in impingement syndrome and chronic pain [8,9]. Previous studies have also evaluated other mechanisms that may influence shoulder muscle fatigue, e.g., alterations in fiber-type distribution [12,13] and different disorders [14].

Surface electromyography (SEMG) is one of the most recognized methods used to investigate the changes in muscle activity in response to muscle-fatigue-induced [12,15,16] injuries to the musculoskeletal system. SEMG is also commonly used to define the appropriate profile of muscle activation and level of contraction [16]. The general model of exercise-induced fatigue has been previously investigated as the effects of static and dynamic exercises on the increase of the amplitude and decrease of the frequency [17]. Fatigue-related evaluation during dynamic tasks is often used during the assessment of daily living activities. However, it has been shown that during dynamic contraction, several factors may interfere with the electromyography (EMG) signal, e.g., changes in the number of active motor units, different lengths of fiber and changes in force and power generation during changes in the range of motion [18]. An increase in the firing rate of the active motor units will occur to compensate for a decrease in the contractility of impaired or fatigued motor units for a given level of force production [19,20]. Changes in the median frequency (MF) might be a sensitive indicator for the objective defining of fatigue [21] and explained as synchronization of the motor units [22].

Shoulder muscle fatigue protocols have been investigated during different tests, e.g., isometric endurance contractions [16], isometric shoulder elevation [12,21,23] as well as isokinetic internal and external rotation protocols [1,2,24] with the use of an isokinetic dynamometer. Dale et al. [1] tested the internal and external rotation of baseball pitchers at 300°/s (12 concentric and eccentric contraptions) followed by 60 pitches and showed greater eccentric internal rotation work-fatigue. As reported by Gaudet et al. [24], isokinetic fatigue testing at velocities 60°/s and 240°/s (three reps with 30 s rest of concentric and eccentric internal and external rotation contractions) demonstrated that velocity had a significant effect on the pectoralis and middle trapezius. However, a second study prepared by Gaudet et al. [24] showed that one set per 50 reps of external and internal rotation at 240°/s may cause a decrease in the peak torque of external and internal rotation, as well as a decrease in MF for the pectoralis, middle deltoid, upper, middle and lower trapezius, and the infraspinatus.

The goal of our study was to examine the muscle activity of the shoulder girdle after isokinetic fatigue, which may simulate muscle activities commonly occurring during specific sport-related activities in overhead asymptomatic athletes. We hypothesized that exercise-induced fatigue, reported after an isokinetic protocol, may cause a decrease in MF of the upper trapezius, infraspinatus and deltoid muscles and potentially reduce stability around the shoulder girdle. Understanding how the execution of a motor task changes under the impact of fatigue may limit the potentially injurious role of fatigue during motor performance.

## 2. Materials and Methods

### 2.1. Study Design

This observational case series study involved two repeated EMG signals which were recorded before and after fatigue protocol [3,6]. To avoid the effects of post-exercise recovery, the experimental protocol time was controlled by an additional operator—the time from the end of the fatigue protocol to the beginning of the measurement was less than 30 s while the measuring time took less than 1.5 min. The SEMG was collected on the shoulder girdle muscles on the right (dominant) side in the following order: upper trapezius (UT), infraspinatus (IS) and anterior (DA), and posterior deltoideus (DP). All participants read and signed an informed consent form approved by the Senate Research Ethics Committee (project identification code: 26/2016 approval date: 13 October 2016). The study was conducted according to the principles of the World Medical Association Declaration of Helsinki.

### 2.2. Participants

A group of healthy recreational male overhead athletes (*n* = 24, age 21.75 ± 2.23 years, body height 181.9 ± 6.0 cm, body weight 78.04 ± 7.8 kg, BMI 23.7 ± 1.7 kg∙m^−2^) were recruited voluntarily. Each of the participants was training at least three times a week (5 ± 2 times a week) for two hours per session. However, they all refrained from any intense physical activity in the two days preceding the test. All participants were right-handed and had 5 to 10 years of training experience in disciplines involving overhead actions. Participants did not experience any history of pain or injuries at the shoulder girdle nor in the thorax/scapular region in the year before the study. The inclusion criteria were (1) training experience in one of the overhead sports ≥ 10 years, and (2) participation in volleyball, handball and tennis at the Academic Sports Association. Exclusion criteria consisted of (1) previous shoulder/elbow/wrist trauma, (2) previous shoulder/elbow/wrist operation and (3) previous pain sensations in the shoulder.

### 2.3. Experimental Procedures

#### 2.3.1. Isokinetic Fatiguing Protocol

The Biodex Multi-Joint System 4 Pro (Biodex Medical System Inc., Shirley, NY, USA) was used to perform a concentric shoulder fatigue protocol. The procedures to set up the isokinetic dynamometer took place one week before the protocol. The device was set for shoulder internal/external rotation (90° of range of motion) with 90° of elbow flexion and the arm in abduction (90°) [3,6,25]. The subjects were seated with their back against a chair at the isokinetic dynamometer and supported with belts to avoid trunk or shoulder movement. The right shoulder was placed in the 90/90 position (degrees of shoulder abduction and elbow flexion respectively). The range of motion was set from 0° (internal rotation) to 90° (external rotation). The fatigue protocol consisted of three sets of 32 repetitions at an isokinetic speed of 120°/s, with a one-minute rest break between sets, as previously defined by Mullaney and McHugh [26]. In particular, the isokinetic speed was set at 120°/s to avoid the inability of reaching peak torque [26] and to increase the tendon loading [27,28]. Prior to data collection, the subjects performed a warm-up which consisted of 10 maximal repetitions at 120°/s. Afterward, the subjects had a five-minute rest and then began the fatigue protocol.

The test was performed with verbal encouragement to make sure that participants generated their maximal effort. We computed torque and the ER/IR ratio as the agonist–antagonist strength indicator, defined as the ratio between the peak torque of external (ER) and internal (IR) rotation (ER/IR ratio) of the shoulder [29]. Changes in the isokinetic torque were defined as the average torques for the first (T_INI_) and last (T_FIN_) three repetitions of each set. Furthermore, the analysis of the torque was performed using the following modes: 1–5 vs. 26–32 vs. 33–37 vs. 60–64 vs. 65–69 vs. 92–96) [26].

#### 2.3.2. EMG Data Collection

Bipolar, disposable, pre-gelled Ag/AgCl surface electrodes with a 20 mm distance between electrode centers were placed on the bellies of the right DP, DA, IS and UT muscles. The electrodes were placed unilaterally on the dominant (right) side. Before the EMG collection, each participant’s skin over the tested muscles was shaved where needed, rubbed with abrasive skin prep and cleaned with alcohol to improve the electrode–skin contact and minimize skin impedance. The electrodes were placed on the (1) upper trapezius approximately 20% medial to the midpoint between the acromion and the C7 vertebra, (2) infraspinatus at the infraspinatus fossa, two finger breadths below the medial portion of the spine of the scapula, (3) deltoideus anterior halfway between the lateral 1/3 of the clavicula and the insertion of the deltoideus, and the (4) deltoideus posterior approximately 2 cm below the lateral border of the spine of the scapula and angled obliquely to the arm [16,30,31]. The exact placement of the electrodes followed the recommendations by Surface Electromyography for the Non-Invasive Assessment of Muscles [32]. The reference electrode was placed over the C7 vertebra. Crosstalk was minimized by the careful placement of electrodes parallel to the muscle fibers based on standard anatomic criteria.

Signals were analog, filtered at 10–500 Hz (Filter FIR, frame 79 points, Type Bandpass, Low frequency 500 Hz, High frequency 10 Hz), amplified 2000 times and sampled at 2000 Hz using a hardware system (TeleMyo 2400 G2, Noraxon U.S.A. Inc., Scottsdale, AZ, USA). EMG signals were recorded while subjects performed the fatiguing protocol, consisting of three sets of 32 internal and external rotations of the right dominant shoulder (all were right) at an angular velocity of 120 deg per second. Signals were acquired online and stored by MyoReaserch XP Master Edition 1.08.32 software (Noraxon U.S.A. Inc., Scottsdale, AZ, USA) installed on a personal computer for offline analysis.

#### 2.3.3. Muscle Fatigue Assessment

**Figure 1:**
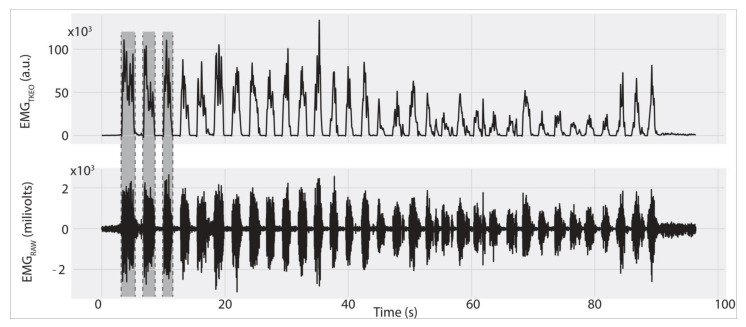
The EMG data analysis during the fatiguing protocol. (EMG_TKEO—_electromyography using the Teager Kaiser energy operator—related in arbitrary units—a.u.).

All data analysis was performed in Python (Python 3.0, Python Software Foundation, Willmington, DE, USA) and the interactive Python integrated development environment [33] using a custom-written code. The recorded EMG (EMG_RAW_) signals were first visually inspected for possible artifacts, then demeaned and band-pass filtered between 10 and 400 Hz with the fourth-order, zero-lag, Butterworth filter. Next, the EMG signals were conditioned, following a highly effective method used to facilitate EMG burst detection, previously described in detail [17,34]. In brief, EMG_RAW_ signals were conditioned using the Teager Kaiser energy operator (EMG_TKEO_), then full-wave rectified and low-pass filtered at 50 Hz to create an EMG envelope (EMG_ENV_) [17,34]. Next, an interactive graphical interface was designed to display EMG_ENV_ signals on the computer screen. An individual with expertise in EMG data analysis used a computer mouse to accurately identify the timing of the first three and the last three EMG bursts recorded during the fatiguing protocol (Figure 1).

#### 2.3.4. Signal Analysis

The manually identified EMG onsets and offsets were then used to compute the median frequency (MF) for the selected EMG bursts from the EMG_RAW_ signals (Figure 1, bottom panel). MF values of the first three EMG bursts were averaged to estimate the initial MF (MF_INI_). Similarly, final MF (MF_FIN_) values were calculated as the average of the last three EMG bursts recorded at the end of the fatiguing protocol. The test lasted approximately six-minutes including rest breaks. The computed MF was divided into 10 equal segments corresponding to 0–100% of the endurance time with 10% increments [16]. The muscle fatigue was quantified as the difference between the MF_INI_ and MF_FIN_ values. The resultant difference, *Δ*MF, was negative when the MF of the EMG signals drifted over time toward lower values, indicating the effect of muscle fatigue. ΔMF was computed for all recorded muscles (i.e., ΔMF_DP_, ΔMF_DA_, ΔMF_IS_, and ΔMF_UT_ for DP, DA, IS and UT muscles, respectively).

#### 2.3.5. Data Analysis

G*Power software (version 3.1.9.2; Kiel University, Kiel, Germany) was used to estimate the required sample size, setting a minimum expected effect size (Cohen’s *f*) of 0.6, an α level of 0.05 and a power (1 − β) of 0.9. The procedure returned a minimum number of 19 participants, with 24 participants recruited to account for potential dropouts.

All descriptive statistics are reported in the text, with figures as means and standard errors unless stated otherwise. We used a two-way repeated-measures ANOVA (RM-ANOVA) to test the effect of *Direction of contraction* (IR, ER) and *Set* (Set 1, Set 2, Set 3) for initial and final torque. A repeated-measures ANOVA was used to calculate the effect of *Time* (MF_INI_ vs. MF_FIN_) across all muscles. To test our main hypothesis that individual muscles will respond differently to a fatiguing task, one-way ANOVA tested the effect of *Muscle* (four levels: DP, DA, IS and UT) on *Δ*MF in the fatiguing tasks. We validated the assumption of the equality of variance using the Levene test. Post-hoc tests with Tukey’s *p*-value adjustment for multiple comparisons were run to explore all significant effects. All statistical tests were performed using Python code and Pingouin library [35], with the significance level set at 0.05.

## 3. Results

Overall, all subjects were able to finish all the sets in the fatiguing task successfully, and all the EMG signals were successfully recorded and analyzed.

### 3.1. Isokinetic Measurements

**Figure 2:**
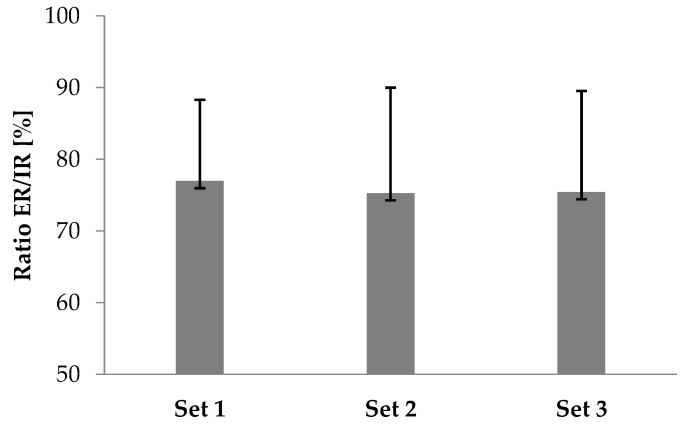
Ratio of external (ER) and internal (IR) rotation (ER/IR ratio) during the isokinetic fatigue protocol.

The two-way RM-ANOVAs revealed a statistically significant *Direction of contraction* × *Set* interaction effect on T_INI_ (F_(1,32)_ = 35.02; *p* = 0.001) and T_FIN_ (F_(1,32)_ =2 5.10; *p* = 0.001). In IR, T_INI_ and T_FIN_ were significantly greater than in ER (*p* ≤ 0.001). Furthermore, T_INI_ decreased significantly by 10.5% and 13.7% during IR (F_(1,32)_ = 13.85; *p* = 0.032) and decreased by 15.2% and 19.9% during ER (F_(1,32)_ = 31.1; *p* = 0.0001). The fatigue effort test progressed to a decrease of T_FIN_ by 12.3% and 14.9% during ER (F_(1,32)_ = 35.1; *p* = 0.0001) (Table 1). Non-significant differences were found in the ER/IR Ratio between each set (*p* ≥ 0.05) (Figure 2).

### 3.2. Muscle Fatigue

**Figure 3:**
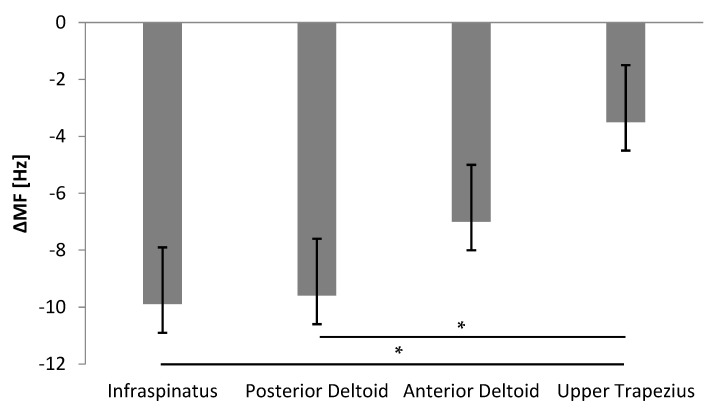
Difference in the median frequency (ΔMF) (Hz) of each muscle in all subjects after the fatigue protocol. (* Significant differences for effect of Muscle on ΔMF in the fatiguing tasks).

All investigated muscles showed signs of muscle fatigue after three sets of 32 internal and external rotations. Average *Δ*MF values consistently dropped after the fatiguing tasks across all recorded muscles, with MF_INI_ = 65.1 ± 1.1 Hz and MF_FIN_ = 57.9 ± 0.9 Hz, and the main effect of *Time* was significant (F_(1,22)_ = 43.15, *p* < 0.001). The significant *Muscle* effect (F_(3,88)_ = 3.74, *p* = 0.014) confirmed these results. The post-hoc tests revealed that, at the end of the fatiguing task, only *Δ*MF values for IS and DP were significantly larger from UT (*p* = 0.002 and *p* = 0.009, respectively) (Figure 3).

## 4. Discussion

This current study is a continuation of our previous studies regarding the muscle morphological and mechanical properties [3] and kinematics [6] of the shoulder girdle. The nobility of this current study is to provide further information about the effect of isokinetic fatigue on shoulder girdle muscle activity. Our results showed a decrease in MF in the examined shoulder girdle muscles, especially in IS and DP after the isokinetic fatiguing protocol. However, IS and DP showed larger *Δ*MF values than UT. Concerning fatigue and the main effect of time, small changes were observed in the MF of DA and UT. The results of our study are in line with the hypothesis, which predicts a decrease in MF of shoulder muscles after a fatiguing protocol. Furthermore, the statistical analysis of the sample size showed that a minimum number of 19 participants was required for evaluation in this research protocol; however, 24 participants were recruited to account for potential dropouts.

Peak torque and fatigue mode (expressed as average torque production for the first five and last five concentric contractions) in both internal rotation and external rotation decreased as the number of repetitions and sets completed increased. Mullaney and McHugh [26] reported decreases in peak torque (25% in internal rotation, 24% in external rotation and 24.8% in internal and external rotation, respectively). In the current study, peak torque decreased by 12.3% and 10.2% (internal and external rotation, respectively). A previous investigation confirmed our observations regarding muscle fatigue development during the experimental protocol and found greater internal rotation peak torque in overhead athletes [29]. The fatigue mode might be an extremely interesting indicator for showing increasing fatigue. In this study, decreases in internal and external rotation were observed during the first five contractions, as well as during the last five contractions for external rotation. These observations are similar to those of Mullaney and McHugh [26]. The ER/IR ratio describes the balance between muscle groups of the shoulder. Previous studies have shown similar results after fatigue [29,35] as in this current study.

Several studies assessed changes in muscle activity and recruitment of the shoulder girdle after internal and external isokinetic protocols in athletes and healthy active subjects [1,2,4], as well as in individuals with impingement syndrome [36,37]. It should be noted that most of the analyzed muscles, i.e., UT, IS and DP, are slow-twitch muscles (type I) [38,39,40] However, AD is a fast-twitch muscle (type II) [40]. According to our results, we found that all investigated muscles showed signs of muscular fatigue after isokinetic fatigue protocol. The highest decrease in MF was reported in IS and DP; however, DA and UT demonstrated smaller changes in MF.

In our study, we observed significant differences in within-group changes in the EMG MF, demonstrating the decrease of MF_FIN_ in all examined muscles after three sets of 32 internal and external rotations. Moreover, we observed a decrease in MF of all muscles, especially IS and DA. Gaudet et al. [24] reported that isokinetic internal and external rotation protocol at 240°/s may lead to a decrease in MF for pectoralis, middle deltoid, upper, middle and lower trapezius and infraspinatus. However, Gaudet et al. [24] found that a significant effect of the isokinetic fatiguing protocol was to decrease the activity of the pectoralis and middle trapezius muscles during an increasing velocity speed protocol, using two different protocols (3 reps with 30 s rest) at 60 and 240°/s. Our findings are consistent with those presented in previous studies [2,4] and confirm those repetitive overhead-related fatigues [1,8,10,11]. Our research protocol included an isokinetic speed of 120°/s, as previously defined by Mullaney and McHugh [26]. Moreover, the isokinetic speed was set at 120°/s to avoid the inability to reach peak torque [26] and increase loading on the rotator cuff tendons [27,28].

Isokinetic exercise-induced fatigue consisting of ER and IR alters muscular properties including timing and co-activation. According to previous studies, the decrease in EMG MF in rotator cuff and shoulder girdle muscles might affect the stabilization of the scapula [2,4]. During increased fatigue, IS and DA might be exposed to excessive shear forces [25]. Furthermore, we have considered the effect of the trapezius muscle on the stabilizing function of the scapula during external rotations. However, DA and IS may directly influence the moment force during external rotation [24]. Our previous study demonstrated that fatigue affected the scapulohumeral rhythm and joints RoM (Range of Motion) in movements in the scapular plane [6]. Acute fatigue causes alterations in the superior translation of the humeral head [41]. Furthermore, it causes scapula reorientation and finally leads to reduction of the subacromial space [42]. Uga et al. [43] showed that isometric external rotation directly contributes to the high activity of the serratus anterior and lower activity of the infraspinatus. The results of our previous studies showed a significant increase in supraspinatus tendon thickness, with a simultaneous reduction in the subacromial space. During reparative internal and external rotation, the fatigue-induced rotator cuff reacted with increased IS stiffness [3]. Moreover, our second study [6] demonstrated alterations in scapulothoracic and glenohumeral mobility as a result of scapular dyskinesis and migration of the humeral head toward the acromion. This mechanism has previously been described as a preliminary factor in impingement syndrome [42]. Moraes et al. [36] found an activation pattern of scapular stabilization during rotation in the following order: upper trapezius, serratus anterior, middle and lower trapezius. However, overhead athletes, e.g., swimmers or throwers, suffer from scapular dyskinesis manifested by inappropriate muscle activation, especially the upper trapezius and serratus anterior [1,29].

Our findings have some potentially practical implications. Our observation is consistent with the findings of our previous studies [3,6], supporting the idea that training loads could be controlled by calibrated rest time between sets of exercise. Moreover, the main findings from this study could be applied in pre-injury and injury prevention programs prepared by physical therapists (e.g., muscle release techniques) and athletic trainers.

Some potential limitations should be considered. First, participants were healthy recreational overhead athletes without previous shoulder injuries and pain sensations. Therefore, it cannot be supposed that similar results would occur in overhead athletics with impingement syndrome or chronic pain. Furthermore, in future studies athletes with more workouts per week should be recruited. Second, the study protocol should include more muscles, e.g., serratus anterior, to assess the contribution for scapular stabilization. Moreover, this study could investigate fatigue after different isokinetic speeds, notwithstanding our study protocol was supported by Mullaney and McHugh [26]. Third, a longitudinal study could infer causation about fatigue alterations in the shoulder girdle. Finally, a wavelets analysis would be better in this case. Future studies should consider those limitations and use additional research tools to investigate the fatigue mechanism of the shoulder girdle.

## 5. Conclusions

The results of our study contribute to the determination of increased fatigue of the shoulder girdle muscles during repeated isokinetic internal-external rotation protocols. We demonstrated a significant decrease in MF in all muscles examined, especially IS and DA. The findings from this study should be applied to further research involving the simultaneous evaluation of morphological and mechanical shoulder girdle properties during muscle activity under conditions of fatigue.

## Figures and Tables

**Table 1:** Torque output for the first three and last three concentric contractions for each set of 32 maximum internal and external rotations.

Torque	Internal Rotation			External Rotation		
	Set 1	Set 2	Set 3	Set 1	Set 2	Set 3
T_INI_ (N·m)	34.04 ± 9.78	30.46 ± 8.61 *	29.38 ± 9.64 *	26.47 ± 6.68	22.44 ± 6.52 *	21.21 ± 6.15 *
T_FIN_ (N·m)	24.94 ± 8.68	23.84 ± 8.57	22.83 ± 7.92	17.87 ± 4.83	15.68 ± 3.89 *	15.21 ± 5.68 *

Torque related in newton-meters (N·m); * Significant values at the *p* ≤ 0.05 level; Abbreviations: T_INI_–torque for the first three repetitions of each set; T_FIN_–torque for the last three repetitions of each set.

## Data Availability

The datasets generated for this study are available on request to the corresponding author.

## References

[1] Dale R.B., Kovaleski J.E., Ogletree T., Heitman R.J., Norrell P.M. (2007). The effects of repetitive overhead throwing on shoulder rotator isokinetic work-fatigue. N. Am. J. Sports Phys. Ther. NAJSPT.

[2] Gaudet S., Begon M., Tremblay J. (2019). Cluster analysis using physical performance and self-report measures to identify shoulder injury in overhead female athletes. J. Sci. Med. Sport.

[3] Klich S., Pietraszewski B., Zago M., Galli M., Lovecchio N., Kawczynski A. (2019). Ultrasonographic and myotonometric evaluation of the shoulder girdle after an isokinetic muscle fatigue protocol. J. Sport Rehabil..

[4] Gaudet S., Tremblay J., Dal Maso F. (2018). Evolution of muscular fatigue in periscapular and rotator cuff muscles during isokinetic shoulder rotations. J. Sports Sci..

[5] Turpin N.A., Martinez R., Begon M. (2020). Shoulder muscle activation strategies differ when lifting or lowering a load. Eur. J. Appl. Physiol..

[6] Zago M., Kawczyński A., Klich S., Pietraszewski B., Galli M., Lovecchio N. (2020). Fatigue-induced scapular dyskinesis in healthy overhead athletes. Front. Bioeng. Biotechnol..

[7] Thigpen C.A., Padua D.A., Michener L.A., Guskiewicz K., Giuliani C., Keener J.D., Stergiou N. (2010). Head and shoulder posture affect scapular mechanics and muscle activity in overhead tasks. J. Electromyogr. Kinesiol..

[8] Chopp J.N., Fischer S.L., Dickerson C.R. (2011). The specificity of fatiguing protocols affect scapular orientation: Implications for subacromial impingement. Clin. Biomech..

[9] Noguchi M., Chopp J.N., Borgs S.P., Dickerson C.R. (2013). Scapular orientation following repetitive prone rowing: Implications for potential subacromial impingement mechanisms. J. Electromyogr. Kinesiol..

[10] Ebaugh D.D., McClure P.W., Karduna A.R. (2006). Effects of shoulder muscle fatigue caused by repetitive overhead activities on scapulothoracic and glenohumeral kinematics. J. Electromyogr. Kinesiol..

[11] Ebaugh D.D., McClure P.W., Karduna A.R. (2006). Scapulothoracic and glenohumeral kinematics following an external rotation fatigue protocol. J. Orthop. Sports Phys. Ther..

[12] Minning S., Eliot C.A., Uhl T.L., Malone T.R. (2007). EMG analysis of shoulder muscle fatigue during resisted isometric shoulder elevation. J. Electromyogr. Kinesiol..

[13] Nussbaum M.A. (2001). Static and dynamic myoelectric measures of shoulder muscle fatigue during intermittent dynamic exertions of low to moderate intensity. Eur. J. Appl. Physiol..

[14] Kinali G., Kara S., Yıldırım M.S. (2016). Electromyographic analysis of an ergonomic risk factor: Overhead work. J. Phys. Ther. Sci..

[15] Stackhouse S.K., Stapleton M.R., Wagner D.A., McClure P.W. (2010). Voluntary activation of the infraspinatus muscle in nonfatigued and fatigued states. J. Shoulder Elbow Surg..

[16] Kawczynski A., Samani A., Mroczek D., Chmura P., Blach W., Migasiewicz J., Klich S., Chmura J., Madeleine P. (2015). Functional connectivity between core and shoulder muscles increases during isometric endurance contractions in judo competitors. Eur. J. Appl. Physiol..

[17] Solnik S., DeVita P., Grzegorczyk K., Koziatek A., Bober T. (2010). EMG frequency during isometric, submaximal activity: A statistical model for biceps brachii. Acta Bioeng. Biomech..

[18] González-Izal M., Malanda A., Navarro-Amezqueta I., Gorostiaga E., Mallor F., Ibanez J., Izquierdo M. (2010). EMG spectral indices and muscle power fatigue during dynamic contractions. J. Electromyogr. Kinesiol..

[19] Cheng A.J., Rice C.L. (2005). Fatigue and recovery of power and isometric torque following isotonic knee extensions. J. Appl. Physiol..

[20] Edwards R., Lippold O. (1956). The relation between force and integrated electrical activity in fatigued muscle. J. Physiol..

[21] Hummel A., Läubli T., Pozzo M., Schenk P., Spillmann S., Klipstein A. (2005). Relationship between perceived exertion and mean power frequency of the EMG signal from the upper trapezius muscle during isometric shoulder elevation. Eur. J. Appl. Physiol..

[22] Semmler J.G. (2002). Motor unit synchronization and neuromuscular performance. Exerc. Sport Sci. Rev..

[23] Wattanaprakornkul D., Cathers I., Halaki M., Ginn K.A. (2011). The rotator cuff muscles have a direction specific recruitment pattern during shoulder flexion and extension exercises. J. Sci. Med. Sport.

[24] Gaudet S., Tremblay J., Begon M. (2018). Muscle recruitment patterns of the subscapularis, serratus anterior and other shoulder girdle muscles during isokinetic internal and external rotations. J. Sports Sci..

[25] Zanca G.G., Oliveira A.B., Saccol M.F., Mattiello-Rosa S.M. (2011). Isokinetic dynamometry applied to shoulder rotators—velocity limitations in eccentric evaluations. J. Sci. Med. Sport.

[26] Mullaney M.J., McHugh M.P. (2006). Concentric and eccentric muscle fatigue of the shoulder rotators. Int. J. Sport Med..

[27] Roy J.S., Ma B., Macdermid J.C., Woodhouse L.J. (2011). Shoulder muscle endurance: The development of a standardized and reliable protocol. Sports Med. Arthrosc. Rehabil. Ther. Technol..

[28] McCreesh K.M., Purtill H., Donnelly A.E., Lewis J.S. (2017). Increased supraspinatus tendon thickness following fatigue loading in rotator cuff tendinopathy: Potential implications for exercise therapy. BMJ Open Sport Exerc. Med..

[29] Batalha N.M.P., Raimundo A.M.d.M., Tomas-Carus P., Fernandes O.d.J.S.M., Marinho D.A., Silva A.J.R.M.d. (2012). Shoulder rotator isokinetic strength profile in young swimmers. Rev. Bras. Cineantropometria Desempenho Hum..

[30] Madeleine P., Farina D. (2008). Time to task failure in shoulder elevation is associated to increase in amplitude and to spatial heterogeneity of upper trapezius mechanomyographic signals. Eur. J. Appl. Physiol..

[31] Madeleine P., Lundager B., Voigt M., Arendt-Nielsen L. (2003). The effects of neck-shoulder pain development on sensory-motor interactions among female workers in the poultry and fish industries. A prospective study. Int. Arch. Occup. Environ. Health.

[32] SENIAM Surface Electromyography for the Non-Invasive Assessment of Muscles; SENIAM Project: 2008. http://www.seniam.org/.

[33] Pérez F., Granger B.E. (2007). IPython: A system for interactive scientific computing. Comput. Sci. Eng..

[34] Solnik S., DeVita P., Rider P., Long B., Hortobágyi T. (2008). Teager–Kaiser Operator improves the accuracy of EMG onset detection independent of signal-to-noise ratio. Acta Bioeng. Biomech..

[35] Guney H., Harput G., Colakoglu F., Baltaci G. (2016). The effect of glenohumeral internal-rotation deficit on functional rotator-strength ratio in adolescent overhead athletes. J. Sport Rehabil..

[36] Moraes G.F., Faria C.D., Teixeira-Salmela L.F. (2008). Scapular muscle recruitment patterns and isokinetic strength ratios of the shoulder rotator muscles in individuals with and without impingement syndrome. J. Shoulder Elbow Surg..

[37] Cools A.M., Witvrouw E.E., Declercq G.A., Danneels L.A., Cambier D.C. (2003). Scapular muscle recruitment patterns: Trapezius muscle latency with and without impingement symptoms. Am. J. Sports Med..

[38] Lindman R., Eriksson A., Thornell L.E. (1990). Fiber type composition of the human male trapezius muscle: Enzyme-histochemical characteristics. Am. J. Anat..

[39] Lovering R.M., Russ D.W. (2008). Fiber type composition of cadaveric human rotator cuff muscles. J. Orthop. Sports Phys. Ther..

[40] Ravn M.K., Ostergaard T.I., Schroeder H.D., Nyengaard J.R., Lambertsen K.L., Frich L.H. (2020). Supraspinatus and deltoid muscle fiber composition in rotator cuff tear conditions. JSES Int..

[41] McClure P.W., Michener L.A., Karduna A.R. (2006). Shoulder function and 3-dimensional scapular kinematics in people with and without shoulder impingement syndrome. Phys. Ther..

[42] Chopp-Hurley J.N., O’Neill J.M., McDonald A.C., Maciukiewicz J.M., Dickerson C.R. (2016). Fatigue-induced glenohumeral and scapulothoracic kinematic variability: Implications for subacromial space reduction. J. Electromyogr. Kinesiol..

[43] Uga D., Endo Y., Nakazawa R., Sakamoto M. (2016). Electromyographic analysis of the infraspinatus and scapular stabilizing muscles during isometric shoulder external rotation at various shoulder elevation angles. J. Phys Ther. Sci..

